# Case Series and Literature Narrative Review of Immune-Mediated Thrombotic Thrombocytopenic Purpura in Children

**DOI:** 10.3390/children13030350

**Published:** 2026-02-28

**Authors:** Letiția-Elena Radu, Andreea Nicoleta Șerbănică, Andra Daniela Marcu, Ana-Maria Bică, Cristina Georgiana Jercan, Radu Obrișcă, Georgiana Gherghe, Gabriela Droc, Dana Tomescu, Anca Coliță

**Affiliations:** 1 Faculty of Medicine, ”Carol Davila” University of Medicine and Pharmacy, 020021 Bucharest, Romania; 2 Pediatrics Department, Fundeni Clinical Institute, 022328 Bucharest, Romania; 3 Hemostasis Laboratory, Fundeni Clinical Institute, 022328 Bucharest, Romania; 4 Anesthesiology and Intensive Care Department, Fundeni Clinical Institute, 022328 Bucharest, Romania

**Keywords:** thrombotic thrombocytopenic purpura, pediatric patients, immune-mediated TTP, ADAMTS13, caplacizumab

## Abstract

**Background/Objectives**: Immune-mediated thrombotic thrombocytopenic purpura (iTTP) is a rare but life-threatening thrombotic microangiopathy in children. Secondary forms, occurring in association with immune dysregulation, autoimmune disease, or other triggers, are particularly challenging to diagnose and manage, and pediatric-specific data remain limited. This study aimed to describe the clinical characteristics, diagnostic pathways, and management of pediatric iTTP and to contextualize these findings within the recent literature. **Methods**: We conducted a retrospective case series of pediatric patients diagnosed with iTTP at a tertiary referral center, between November 2021 and January 2026. Clinical presentation, laboratory findings, including ADAMTS13 activity and ADAMTS13 inhibitors, associated conditions, treatment strategies, and outcomes were reviewed. In parallel, a narrative literature review was performed focusing on pediatric immune-mediated secondary TTP published over the past five years. **Results**: Four pediatric patients (three females, one male; median age 14 years) met inclusion criteria. All presented with severe thrombocytopenia and microangiopathic hemolytic anemia, accompanied by prominent neurologic manifestations in three cases. Severe ADAMTS13 activity deficiency (≤10%) with positive inhibitors was documented in all patients. Secondary iTTP occurred in association with evolving systemic autoimmunity, systemic lupus erythematosus, common variable immunodeficiency, or without an identifiable trigger at presentation. High clinical probability scores facilitated early diagnosis. Management required plasma exchange, corticosteroids, and targeted and immunomodulatory therapy. **Conclusions**: Pediatric secondary iTTP is a heterogeneous condition that frequently presents with diagnostic ambiguity and severe neurologic involvement. Early recognition, prompt initiation of TTP-directed therapy, and comprehensive immunologic evaluation are critical for favorable outcomes. Case series combined with narrative reviews remain valuable for advancing understanding and optimizing individualized care in this rare pediatric disorder.

## 1. Introduction

Thrombotic thrombocytopenic purpura (TTP) is a rare, but potentially life-threatening thrombotic microangiopathy (TMA) characterized by severe thrombocytopenia, microangiopathic hemolytic anemia, and variable organ ischemia resulting from disseminated platelet-rich thrombi in microcirculation [1,2,3,4]. The central pathophysiological mechanism is a severe deficiency of the von Willebrand factor (vWF)–cleaving protease ADAMTS13, leading to the accumulation of ultra-large vWF multimers and uncontrolled platelet aggregation under high-stress conditions [3,4,5].

In children, TTP represents an exceptionally uncommon diagnosis, accounting for a small fraction of pediatric TMAs, yet it is associated with significant morbidity and mortality if diagnosis and treatment are delayed [6,7]. Pediatric TTP may be congenital (cTTP), due to biallelic pathogenic variants in the ADAMTS13 gene (namely, Upshaw–Schulman syndrome), or acquired, most often immune-mediated (iTTP), resulting from autoantibodies (Abs) that inhibit ADAMTS13 activity or accelerate its clearance [4,8,9].

Within acquired forms, the distinction between primary or secondary TTP is particularly challenging in children. Secondary TTP refers to cases occurring in association with an identifiable underlying condition or trigger, such as systemic autoimmune diseases, infections, malignancies, organ transplantation, pregnancy, or exposure to certain medications and vaccines [2,10,11,12]. The presence of an underlying disease may obscure the diagnosis, modify the clinical presentation, and influence both treatment response and prognosis [6,7]. In pediatric practice, these associations are heterogeneous and often overlap with clinical contexts in which alternative TMAs—such as hemolytic uremic syndrome (HUS), macrophage activation syndrome, or disseminated intravascular coagulation (DIC)—are more commonly suspected [6,8]. Autoimmune disorders, particularly systemic lupus erythematosus (SLE), are among the most frequently reported associations in pediatric secondary TTP, but infectious triggers, post-transplant immune dysregulation, malignancies, and, more recently, vaccine-associated cases have also been described [10,11].

Major advances in TTP management over the past decade have resulted from improved diagnostic tools and emerging targeted therapies. Measurement of ADAMTS13 activity and detection of ADAMTS13 inhibitors or specific autoAbs are now the focus of diagnostic confirmation, risk stratification, and therapeutic decision-making [1,8,9]. Furthermore, caplacizumab and B-cell–directed immunosuppressive therapies have significantly improved outcomes in iTTP, reducing early mortality, accelerating platelet recovery, and lowering relapse rates [12,13,14,15,16].

The true incidence of secondary TTP in children is unknown, and evidence-based guidance regarding optimal management —especially concerning the timing and intensity of immunosuppression— remains poorly defined. Over the past five years, international guidelines, narrative reviews, and registry-based analyses have refined the conceptual framework of iTTP and highlighted important unmet needs in pediatric care [1,2,6]. Nevertheless, secondary forms of pediatric TTP are often grouped together with primary iTTP or excluded from analyses, limiting the applicability of existing recommendations to this vulnerable population.

Given the rarity of the condition and the heterogeneity of associated triggers, case series combined with narrative literature reviews remain a valuable approach for advancing knowledge in pediatric secondary TTP. Such studies allow for detailed clinical characterization, exploration of diagnostic pathways, assessment of treatment strategies, and identification of outcome patterns that may not be captured in predominantly adult cohorts [7,15,17,18].

The aim of the present paper is to report a series of pediatric patients with iTTP, illustrating diverse clinical contexts, and to provide a narrative review of the recent literature on the subject. By integrating our clinical experience with published evidence, we seek to highlight key diagnostic challenges, discuss pathophysiological considerations, and review current management strategies and outcomes.

## 2. Materials and Methods

### 2.1. Study Design and Setting

This study was designed as a retrospective case series combined with a narrative literature review. The case series includes pediatric patients diagnosed and treated for iTTP at Fundeni Clinical Institute, Bucharest, Romania, from November 2021 until January 2026. Clinical, laboratory, and therapeutic data were extracted from medical records.

The narrative literature review was conducted to contextualize the presented cases within current evidence on pediatric iTTP published over the last five years.

### 2.2. Patient Selection

Pediatric patients (aged <18 years at diagnosis) were eligible for inclusion in the case series if they met all of the following criteria: (1) clinical and laboratory features consistent with TMA, including thrombocytopenia and microangiopathic hemolytic anemia; (2) severe ADAMTS13 activity deficiency (≤10%), with detectable anti-ADAMTS13 inhibitors; (3) availability of sufficient clinical and laboratory data to allow detailed case description.

Patients with genetically confirmed cTTP were excluded. Cases in which an alternative diagnosis of thrombotic microangiopathy (e.g., Shiga toxin–associated hemolytic uremic syndrome, complement-mediated TMA, DIC) was considered more likely were also excluded.

### 2.3. Data Collection

For each patient, the following data were collected: demographic characteristics (age, sex); underlying disease or triggering condition associated with secondary TTP; clinical presentation, including neurological, renal, gastrointestinal, and other organ involvement; laboratory parameters at diagnosis and during follow-up, including complete blood count, markers of hemolysis, coagulation profile, renal function, and inflammatory markers; ADAMTS13 activity levels; ADAMTS13 inhibitors by mixing studies; next-generation sequencing (NGS) test, when available; therapeutic interventions, including plasma exchange (PEX) or plasma infusion, corticosteroids, immunosuppressive agents (e.g., methyl prednisolone, rituximab, mycophenolate mofetil), and targeted therapy (e.g., caplacizumab); clinical course, response to therapy, complications, exacerbation or relapse, and outcome.

Already established scores were used to assess the probability of TTP [19,20]:

(1) The PLASMIC score:
  - platelet count <30,000/mmc: 1 point;
  - hemolysis signs (reticulocytosis, low haptoglobin, elevated bilirubin): 1 point;
  - creatinine level <2 mg/dL: 1 point;
  - mean corpuscular volume (MCV) <90 fl: 1 point;
  - international normalized ratio (INR) <1.5: 1 point;
  - absence of active cancer: 1 point;
  - absence of post-transplant status: 1 point.

A high risk (6–7 points) suggests the diagnosis of TTP very possible and treatment should be started immediately, intermediate risk (5 points) warrants a close observation and low risk (0–4 points) implies ADAMTS13 depletion unlikely.

(2) The French score:
  - platelet count <30,000/mmc: 1 point;
  - creatinine level <2.26 mg/dL: 1 point;
  - fever or neurological symptoms: 1 point;
  - absence of active cancer and transplant status.

A score of 0 or 1 represents a low probability of TTP, while a score of 2 or 3 marks a high probability.

### 2.4. ADAMTS13 Activity Testing

Accurate and rapid determination of ADAMTS13 activity is essential for the diagnosis and therapeutic management of TTP. Among available methodologies, the fluorescence resonance energy transfer (FRET)-based assay using the FRETS-VWF73 substrate is widely accepted due to its high sensitivity, specificity, and suitability for routine clinical laboratory use. The assessment of ADAMTS activity was performed in-house, in Fundeni Clinical Institute, Hemostasis Laboratory, using The Ceveron^®^ 100-Series automatic machine. Both reagents and machine were produced by Technoclone Herstellung von Diagnostika und Arznelmitteln GmbH, Vienna, Austria.

Results were interpreted using Ceveron Software v 3.0, and were expressed as a percentage of activity relative to normal pooled plasma, which was defined as 100% activity. ADAMTS13 activity was interpreted as follows: normal activity (50–150%), moderately reduced activity (10–50%), severely reduced activity (<10%, highly suggestive of TTP).

### 2.5. Definitions

– Secondary TTP = an iTTP occurring in temporal association with an identifiable underlying condition or trigger, in the absence of congenital ADAMTS13 deficiency;
– Severe ADAMTS13 deficiency = an activity ≤10%;
– Clinical response = a sustained normalization of platelet count (≥150,000/mmc) and lactate dehydrogenase (LDH) <1.5 × normal value, plus no new ischemic organ injury and resolution of hemolysis;
– Clinical remission = a sustained clinical response with no treatment ≥30 days or until ADAMTS13 activity ≥20%;
– Clinical exacerbation = a recurrence of thrombocytopenia +/− new ischemic injury after achievement of clinical response and before clinical remission;
– Clinical relapse = a recurrence of thrombocytopenia +/− new ischemic injury after achievement of clinical remission [21,22].

### 2.6. Literature Review Strategy

A narrative literature review was conducted using PubMed and Scopus databases. Search terms included “immune thrombotic thrombocytopenic purpura”, “secondary thrombotic thrombocytopenic purpura”, “pediatric thrombotic thrombocytopenic purpura”, “ADAMTS13”, and “caplacizumab”. Peer-reviewed articles published in English between 2020 and 2025 were analyzed. Given the rarity of pediatric secondary iTTP, adult studies were also included, particularly for pathophysiology and therapeutic strategies. Due to the heterogeneity of published data, a narrative rather than systematic approach was chosen to allow integration of diverse evidence sources and detailed comparison with the presented cases.

### 2.7. Ethical Considerations

This study was conducted in accordance with the principles of the Declaration of Helsinki. Due to the retrospective nature of the case series and the use of anonymized data, formal informed consent and ethical committee approval were waived for this paper. The legal guardians signed informed consent forms for both diagnostic and treatment procedures, and for participating in medical scientific activity, as per each hospital admission, due to local procedures.

ChatGPT v 5.2 was used for superficial text editing (e.g., grammar, spelling, punctuation) and to create the 4 figures.

## 3. Results

Four patients met the inclusion and exclusion criteria for iTTP. The summarized version of the diagnostic criteria is represented in Table 1.

### 3.1. Patient 1

A 14-year-old female patient presented according to the clinical information and paraclinical results found in Table 1.

Additional laboratory data: normal immature platelet fraction (IPF), no blasts or dysplasia, elevated D-Dimers, fibrinogen slightly decreased, low C3 and C4, negative antinuclear and antiphospholipid Abs, positive nucleosome and double-stranded DNA Abs (>200 U/mL, 70.4 U/mL respectively), positive lupic cells, no inflammatory syndrome, no acute viral or bacterial infections. Even though she did not meet the EULAR criteria due to negative antinuclear Abs, the clinical and laboratory data suggested an on-going autoimmune disorder. No genetic testing was performed.

**Figure 1:**
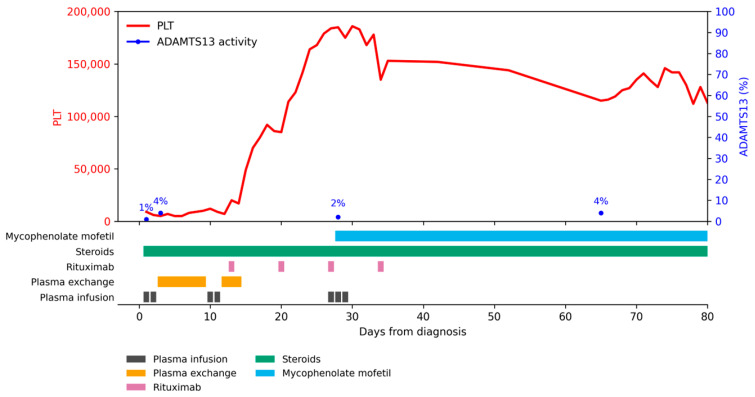
Disease evolution for Patient 1. PLT = platelets (×10^9^/mmc).

Treatment course and follow up (Figure 1): plasma infusions (days 1–2, 10–11), PEX (days 3–9 12–14), high-dose intravenous methylprednisolone for 5 days with slow tapering during the subsequent two months, rituximab (375 mg/m^2^) (days 13, 20, 27, 34). At that time, caplacizumab was not available in our country for pediatric patients. An exacerbation occurred with thrombosis of the right ilio-femoral–popliteal venous space, plasma infusions and mycophenolate mofetil were added at that time. A second exacerbation happened on day 65, which required 3-day high-dose methylprednisolone, with tapering thereafter. Clinical remission was obtained after day 120 (March 2022); interestingly, ADAMTS13 activity remained decreased until May 2025 (107%).

### 3.2. Patient 2

A 14-year-old female patient presented according to the clinical information and paraclinical results found in Table 1.

Considering the medical history (namely, cellulitis of the right upper lower limb), and decreased IgA fraction, an NGS genetic test (Blueprint Genetics, FLEX Primary Immunodeficiency Panel Plus, 451 gene mutations) was performed, which revealed three genetic alterations:

(1) *c.204dup*, p.(Leu69Thrfs*12), a mutation in the TNFRSF138 gene, associated with common variable immunodeficiency (CVID), classified as pathogenic.
(2) *c.554T>G*, *p.(Phe185Cys)*, a second mutation in the TNFRSF138 gene, classified as pathogenic.
(3) *c.2351G>A*, *p.(Arg784Gln)*, a heterozygote mutation in the ADAMTS13 gene, classified as variant of uncertain significance.

Therefore, the final diagnosis for this patient was an acute episode of iTTP, secondary to CVID.

**Figure 2:**
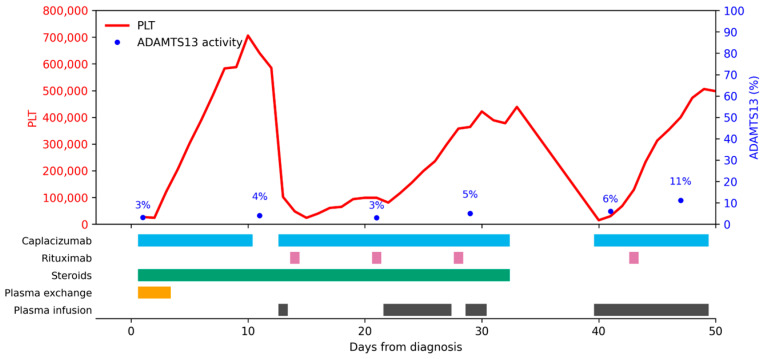
Disease evolution for Patient 2. PLT = platelets (×10^9^/mmc).

Treatment course and follow up (Figure 2): three daily PEX, oral methylprednisolone (1.5 mg/kg/day) for 10 days, with slow tapering for one month, caplacizumab (10 doses; at that time, it was available only off-label for children). A clinical response was obtained on day 5. The patient experienced an exacerbation 48 h after; plasma infusions and rituximab (375 mg/m^2^) on days 14, 21, 28 were given, with a second clinical response on day 25. Treatment was stopped on day 33, and one week later, a second exacerbation occurred. Caplacizumab was resumed for an additional 10 days, and plasma infusions and a fourth rituximab dose were administered. Clinical response was evident on day 45, and therapy was stopped on day 49. Clinical remission was achieved in November 2025, with 62% ADAMTS13 activity.

### 3.3. Patient 3

A 17-year-old female patient presented according to the clinical information and paraclinical results found in Table 1.

Additional laboratory data: slightly low C3 and C4, high levels of antinuclear, antiphospholipid, nucleosome and double-stranded DNA Abs, no evidence of acute infection. The thoracic scan showed pericarditis and small pleural effusion with passive pulmonary collapse. She was also tested by the NGS method (Blueprint Genetics, FLEX Primary Immunodeficiency Panel Plus, comprising 416 gene mutations), with a negative result for underlying immunodeficiency or genetic predispositions. Therefore, the final diagnosis was acute iTTP, secondary to SLE.

**Figure 3:**
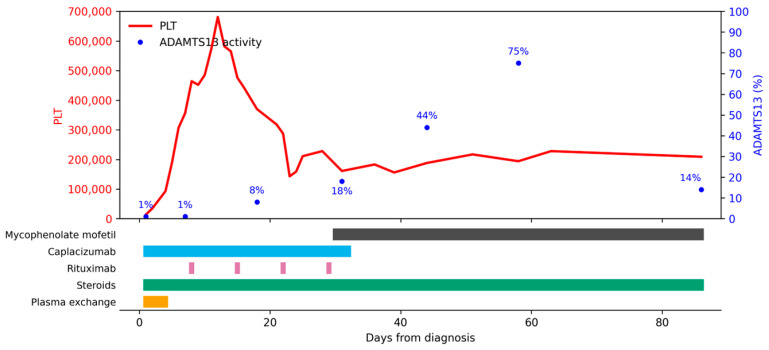
Disease evolution for Patient 3. PLT = platelets (×10^9^/mmc).

Therapeutic course and follow up (Figure 3): PEX (days 1–4), caplacizumab (32 doses), methylprednisolone (1.5 mg/kg/day) (14 days, with slow tapering for two months). Rituximab (375 mg/m^2^) (days 8, 15, 22, 29), mycophenolate mofetil. Clinical response was achieved on day 7. The ADAMTS13 activity level was 18% on day 31. Treatment was discontinued in August 2025. Almost one month later, the patient experienced partial thrombosis of the left femoral vein, concurrent with normal platelet count, but a transitory decline in ADAMTS13 activity (14%) with spontaneous resolution after approximately 60 days (114%). Low-molecular-weight heparin was given, and all disease-directed therapy was discontinued gradually. She is in clinical remission.

### 3.4. Patient 4

An 11-year-old male patient presented according to the clinical information and paraclinical results found in Table 1.

Additional laboratory findings: azotemia, elevated pancreatic enzymes, slightly increased inflammatory markers, no evidence of acute infection, normal C3, C4, antinuclear Abs, double-stranded DNA Abs, and immunoglobulin fractions. Abdominal ultrasound showed hepatomegaly and bilateral renal enlargement, with unexceptional chest Xray.

**Figure 4:**
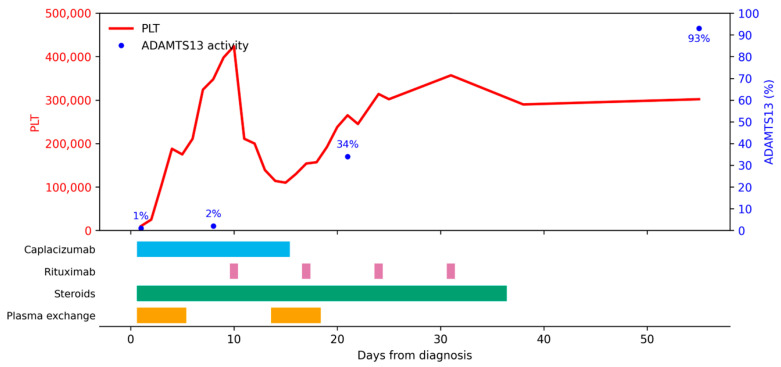
Disease evolution for Patient 4. PLT = platelets (×10^9^/mmc).

The patient received the following: PEX (days 1–5), caplacizumab (15 days, stopped due to shortage), and intravenous methylprednisolone (1.5 mg/kg/day) (14 days, with ongoing oral tapering). Even though clinical response was obtained on day 5, one week later, he experienced a short paroxysmal tonic episode with loss of consciousness, with normal subsequent neurological examination and unremarkable cerebral imaging, and a drop in platelet count. Due to the exacerbation of the disease, the patient received PEX (4 days) and four weekly doses of rituximab (375 mg/m^2^). The ADAMTS13 activity level was 34% on day 21, and 101% on day 31. Currently, he maintains his clinical response (Figure 4).

## 4. Discussions/Narrative Review of the Literature

In children, iTTP is a rare but life-threatening thrombotic microangiopathy, in which delayed recognition may result in irreversible organ injury or death. The present cases illustrate the heterogeneity of pediatric iTTP and highlight several diagnostic and therapeutic challenges that are consistently reported in the literature but remain insufficiently addressed in pediatric-specific guidelines.

The following narrative review synthesizes recent evidence on immune-mediated secondary TTP with a focus on pediatric-specific considerations. Emphasis is placed on diagnostic ambiguity, immune-dysregulation–related triggers, and therapeutic decision-making, reflecting the challenges encountered in a clinical setting.

### 4.1. Incidence and Epidemiology

TTP is an uncommon TMA across all age groups, with a substantially lower incidence in the pediatric population than in adults. Contemporary epidemiologic syntheses and guidelines emphasize that robust incidence estimates are largely derived from adult registries and multicenter cohorts, while pediatric incidence is inferred from smaller series and retrospective datasets. This imbalance is particularly relevant for secondary iTTP, where the rarity of cases, referral bias, and diagnostic overlap with other pediatric TMAs (especially HUS and infection-associated TMAs) may lead to under-recognition and under-reporting [1,2,3,4,6,7,8,21,22]. Real-world datasets from the United States and other regions describe contemporary clinical and therapeutic pathways, the health-system impact of resource use, and outcomes in adult cohorts treated with modern combinations; while not pediatric-specific, they support extrapolated trajectories and treatment intensification options [23,24,25,26]. Available pediatric evidence is limited to case reports and small series, indicating that iTTP in children is substantially rarer than in adults and is typically encountered in tertiary referral centers [25,27,28]. Consequently, epidemiologic statements in children must remain descriptive rather than quantitative.

A key pediatric diagnostic pitfall is to discriminate among different TMAs, especially HUS or sepsis-associated thrombocytopenia, particularly when neurologic symptoms dominate or when renal impairment is present. In addition, vitamin B12 deficiency-related metabolism-mediated TMA is highlighted as an important mimic that can resemble TTP; the absence of reticulocytosis is key to recognizing such a diagnosis and clinically relevant for avoiding unnecessary PEX and for appropriate therapy [29]. Pediatric-focused reviews explicitly address these challenges and recommend systematic TMA evaluation algorithms with early consideration of ADAMTS13 testing and prompt TTP-directed treatment with multidisciplinary critical care support, when clinical probability is high [22,27,30,31,32].

Within pediatrics, age at presentation and recurrence patterns help a correct diagnosis. Thus, cTTP is more likely in neonates/infants and in children with recurrent episodes without a clear immune trigger, whereas iTTP is reported more often in older children and adolescents. The median age of the index iTTP episode is estimated at 12 years and the sex ratio is ~2–2.5/1 female/male. Although the first case of iTTP was described in 1924 by Eli Moschcowitz in a 16-year-old girl, the incidence of pediatric patients is only 4–10% of all iTTP and comprises approximately two-thirds of all TTP in children. Around 56% of pediatric onset iTTP cases remain idiopathic [6,7,8,21,22,33,34].

These trends are consistent with our cohort, in which three of four patients were adolescent females aged 14–17 years, while the youngest patient (Patient 4) presented at 11 years of age. In our series, Patients 2 and 4 initially underwent evaluation for neurologic or other immune-mediated conditions unrelated to TTP, illustrating how epidemiologic rarity and atypical initial presentations can delay disease recognition.

### 4.2. Etiology of Secondary TTP

Secondary iTTP is defined by severe ADAMTS13 functional deficiency in association with an identifiable trigger or underlying condition. Within this reference set, multiple case-based sources emphasize autoimmune and immune-dysregulation contexts, infectious triggers, and iatrogenic immune activation.

In pediatrics, the available literature repeatedly points to evolving immune dysregulation as a dominant theme: children may present with TTP in the setting of incomplete autoimmune phenotypes that do not yet fulfill formal classification criteria. Autoimmune conditions (including SLE, lupus-like disease, and other mixed connective tissue disease), infections, malignancy-related immune perturbations, and post-transplant immune dysregulation are described as relevant contexts, although the distribution differs from adults, where pregnancy-associated immune changes and established autoimmunity are more frequent [1,2,3,7,9,10,11,21,22,28,33,34,35,36,37,38,39,40]. A practical pediatric implication is that “secondary” may remain a provisional label at first presentation. Several pediatric series and case reports emphasize longitudinal follow-up to capture a definable autoimmune disorder, and they highlight that secondary iTTP can represent the first major clinical expression of systemic autoimmunity, rather than after longstanding autoimmunity [41].

Additional case-based studies discuss overlap with immune thrombocytopenic purpura (ITP), underscoring that immune cytopenias can coexist and complicate early diagnostic assessment when thrombocytopenia is the presenting abnormality [41,42]. From a pediatric perspective, there is also a reference regarding a case of anti–glomerular basement membrane disease associated with TTP, which illustrates that secondary iTTP may arise in the setting of organ-specific autoimmunity and nephro-immunologic disease [43].

In parallel, reports of infection- or vaccine-associated iTTP provide plausibility for acute immune activation precipitating anti-ADAMTS13 autoimmunity, reinforcing that acute infections can precipitate a TMA phenotype and that careful diagnostic judgement is required to distinguish infection-associated TMAs. For example, COVID-19 has been reported to induce thromboembolic events, especially in vulnerable patients. Another possible trigger for iTTP is the cross-reaction of some vaccine antigens with ADAMTS13 enzyme [9,10,11,25,27,34,37,38,39,40,44]. Mostly, these data are derived from adults and must be contextualized carefully when extrapolated to children.

Finally, iatrogenic immune-triggered TTP (e.g., checkpoint inhibitor–related) is reported as a rare, but potentially fatal toxicity. It underscores the possibility of abrupt, severe presentations with poor outcomes if not recognized early, reinforcing the need for high clinical suspicion in appropriate contexts [45,46].

The association between secondary iTTP and autoimmune disorders is clearly illustrated by Patients 1 and 3; Patient 3 fulfilled immunologic and clinical criteria consistent with SLE at presentation, with hypocomplementemia, positive antinuclear and antiphospholipid Abs, serositis, and neuropsychiatric involvement; in contrast, Patient 1 presented with severe iTTP in the context of evolving autoimmunity, characterized by high titers of double-strained anti-DNA and anti-nucleosome Abs, but without fulfilling formal EULAR criteria for SLE at onset. These cases underscore that pediatric secondary iTTP may represent an early or sentinel manifestation of systemic autoimmunity, necessitating long-term immunological surveillance even when a definitive autoimmune diagnosis is initially uncertain.

As mentioned above, primary immunodeficiency as a trigger for secondary iTTP is exceedingly rare in children. Patient 2 exemplifies this unusual association, with iTTP occurring in the context of CVID, confirmed by two pathogenic variants in TNFRSF13B and immunoglobulin abnormalities. Although a heterozygous ADAMTS13 variant of uncertain significance was also identified, the presence of ADAMTS13 inhibitors and the absence of biallelic pathogenic variants confirm an immune-mediated etiology. This case highlights the importance of considering broader immune dysregulation—not solely classic autoimmunity—as a predisposing factor for secondary iTTP in pediatric patients.

In Patient 4, no clear autoimmune, infectious, or immunodeficiency trigger was identified at the time of diagnosis, illustrating that secondary iTTP may initially appear idiopathic. Such cases require careful longitudinal evaluation, as an underlying immune-mediated condition may emerge over time.

### 4.3. Pathophysiology and Pediatric-Specific Considerations

Across age groups, the mechanistic hallmark of iTTP is a severe functional deficiency of ADAMTS13, resulting in reduced cleavage of ultra-large vWF multimers and downstream platelet-rich microthrombi in small vessels. The immune-mediated form of TTP is caused by Abs, mainly IgG, which are directed against ADAMTS13, where they inhibit its function or enhance its clearance [33]. Contemporary reviews describe two non-mutually exclusive immune mechanisms: (1) inhibitory autoAbs that block enzymatic activity and (2) non-inhibitory Abs that accelerate ADAMTS13 clearance. Under physiological conditions, ADAMTS13, found in the plasma, circulates in “closed” conformation, making it impenetrable to the two categories of Abs. In contrast, it is found in open conformation during an acute episode of iTTP [47,48,49], becoming vulnerable to the Abs action. The resulting vWF-driven platelet aggregation explains the typical laboratory phenotype (severe thrombocytopenia, microangiopathic hemolysis) and the organ distribution of ischemic injury [3,4,5,9,15,18,39,47]. Available reviews detail the regulation of ADAMTS13 synthesis, secretion, and inhibition by autoAbs, providing a framework for understanding disease heterogeneity [50].

Although severe ADAMTS13 deficiency is necessary for the development of TTP, enzyme deficiency alone may not be sufficient to induce the clinical syndrome. The formation of circulating immune complexes and the activation of complement systems typically occurring via the classical pathway have also been implicated in the pathophysiological mechanism of iTTP, making the “second hit” hypothesis possible. On the other hand, human leukocyte alleles (HLA) are thought to be involved in the development of autoimmune diseases, and studies have indicated that the expression of HLA DQ-7, HLA DRB1*11, and HLA DRB3* may increase the risk of developing iTTP, while HLA DRB1*04/DR53 had a possible protective role [34,39].

Importantly, conceptual work comparing immune and hereditary TTP questions whether ADAMTS13 deficiency alone explains clinical heterogeneity, pointing to modifiers such as endothelial activation, inflammatory milieu, and patient-specific susceptibility [30]. Consistent with this, cytokine profiling studies demonstrate distinct anti-inflammatory cytokine imprints in TTP compared with COVID-19–associated illness, supporting the idea that systemic immune signaling differs across thrombo-inflammatory syndromes, even when thrombocytopenia and organ dysfunction overlap clinically [49,51].

In pediatrics, the pathophysiological core is similar, but clinical expression is shaped by a higher proportion of first-episode disease, fewer vascular comorbidities, and a higher probability of cTTP in younger children. Pediatric-focused papers strongly advocate for definitive classification (immune-mediated vs. congenital) whenever feasible [30,31,32], including molecular diagnosis in suspected congenital cases; however, in many clinical settings, early management decisions must precede complete etiological confirmation. Therefore, serial assessment of ADAMTS13 activity and careful documentation of treatment response are frequently used as pragmatic surrogates while definitive testing is pursued [8,21,22].

All of the patients in our series demonstrated severe ADAMTS13 activity deficiency (≤3.2%) and positive inhibitors (<7%), confirming iTTP. The coexistence of several autoAbs in Patients 1 and 3 further support immune-driven ADAMTS13 dysfunction. In Patient 2, the presence of a heterozygous ADAMTS13 variant likely acted as a modifying factor rather than a primary cause, illustrating how genetic susceptibility and immune dysregulation may converge in pediatric iTTP.

### 4.4. Clinical Manifestations

Adult cohorts confirm wide phenotypic variability, with severe organ failure observed in critically ill patients [52]. Pediatric iTTP commonly presents with abrupt symptoms and prominent neurologic involvement. Classically, a patient with iTTP exhibits thrombocytopenia and microangiopathic hemolytic anemia and may develop neurologic symptoms, renal manifestations, fever, and dysfunction of other organs. In contemporary practice, the full “pentad” is uncommon at presentation; instead, symptom clusters vary by trigger and timing of diagnosis.

Pediatric series describe headache, agitation, confusion, focal deficits, transient ischemic symptoms, and seizures as frequent manifestations; these symptoms may precede recognition of hematologic TMA, especially when early laboratory testing is incomplete. In some studies, neurological manifestations occurred in 73% of TMA patients at diagnosis, and they consisted of severe manifestations [53]. Some cTTP patients can present only with neurologic symptoms and essentially normal platelet count [30]. Mucocutaneous bleeding manifestations (petechiae, ecchymoses, epistaxis, hematuria) are expected consequences of severe thrombocytopenia. In children, renal involvement is often present but tends to be milder than in other TMAs, and severe renal failure is less typical in iTTP than in complement-mediated HUS [6,7,8,21,22,34,40,41,42,43,44].

In our patients, according to the data presented in Table 1, neurologic involvement was a dominant feature of pediatric iTTP and, in some cases, preceded recognition of the underlying hematologic disorder. Headache, agitation, focal neurologic deficits, and altered mental status are frequently reported and were prominent in three of the four patients in our series (Patients 2, 3, and 4). In Patient 2, the initial presentation mimicked meningoencephalitis, while Patient 3 exhibited severe neuropsychiatric agitation and focal deficits, highlighting the risk of misdiagnosis when neurologic symptoms dominate clinical evaluation. Bleeding manifestations related to profound thrombocytopenia were observed in Patients 1 and 4, including petechiae, ecchymoses, and hematuria. Renal involvement was present but mild in all cases, consistent with reports indicating that severe renal failure is less common in iTTP than in other pediatric TMAs. The absence of the full classical pentad in all patients reinforces the contemporary understanding that reliance on complete symptom clusters may delay diagnosis.

### 4.5. Laboratory Investigations and Imaging

Routine laboratory testing typically establishes the TMA phenotype: severe thrombocytopenia, microangiopathic hemolytic anemia (schistocytes), elevated LDH, indirect hyperbilirubinemia, and reduced haptoglobin. Reticulocytosis supports hemolysis-driven anemia. In pediatric reports, inflammatory markers may be variably elevated, particularly when secondary TTP occurs in the setting of infection or evolving systemic autoimmunity; however, these markers are not diagnostic and should not delay TTP-directed therapy when suspicion is high [1,2,6,7,8,21,22,41,44,54]. A negative Coombs test is used to differentiate Evans syndrome, while an unremarkable coagulation panel excludes DIC [35].

In oligosymptomatic patients, distinguishing iTTP from ITP can sometimes be challenging [42,55]. Interestingly, in newly diagnosed iTTP patients, the IPF value is normal or low, suggesting the inability of bone marrow to compensate for peripheral platelet destruction, thus indicating that there could be an additional defect at this level. The hypoproduction of immature platelets is reversed with the initiation of PEX, as soon as after the first procedure; therefore, IPF could prove to be an important and cost-effective marker for these patients [54].

Specialized blood testing involves ADAMTS13. Severe activity reduction (meaning <10 IU/dL or <10% of normal) supports TTP when clinical context is compatible; serial activity testing may also help characterize remission and relapse risk. Where available, ADAMTS13 inhibitor/autoAb testing refines classification, and quantitative ADAMTS13 antigen levels may assist in clinical interpretation. These tests support the diagnosis and inform about the relapse risk and treatment intensity required. The French and the PLASMIC scores are validated and predictive and are frequently used in urgent-care clinical settings. Even though they were not developed to substitute ADAMTS13 activity testing, which is vital for the confirmation and follow-up of TTP, the two scores are able to facilitate the clinical diagnosis of this life-threatening disease [19,20,47,50,56]. There are debates on economic and operational evaluations of ADAMTS13 testing strategies, comparing rapid, in-house, and send-out approaches, analyzing how time to result impacts therapeutic decisions, costs, and, potentially, outcomes by influencing early initiation of targeted therapy and avoiding unnecessary treatments in mimicking TMAs [57].

In addition, an interesting study proposes distinguishing TTP from other causes of anemia and thrombocytopenia using a carbon monoxide CO breath test in a specific clinical context, reflecting ongoing efforts to improve diagnostic accuracy and reduce treatment delay; such approaches are adjunctive and do not replace ADAMTS13-based confirmation where available [58].

Genetic evaluation is critical when cTTP is part of the differential diagnosis, particularly in younger children, recurrent disease, or atypical immune contexts. A study on genetic variants modulating renal function in iTTP suggests that host genetic background can influence organ vulnerability even in immune-mediated disease [59]. Pediatric-focused publications further stress that genetic testing is essential to definitively diagnose cTTP, but this is frequently unavailable at the point of acute care.

Imaging (e.g., CT or MRI of the brain, abdominal ultrasound) is used primarily to evaluate complications or exclude alternative diagnoses, rather than to confirm TTP. Pediatric imaging is therefore best viewed as adjunctive—useful for assessing acute neurologic events, ruling out hemorrhage, and documenting organ involvement, but not replacing hematologic and ADAMTS13-based diagnosis [8,21,22,39,41,43,60]. Notably, imaging studies demonstrate that both overt and subclinical cerebral ischemic lesions are common, particularly in adults [53,60].

It is worth mentioning that identifying the trigger for or the underlying cause of the index episode is important for a complete and accurate diagnosis, but also for long-term follow-up. Predisposing conditions are established in 27–69% of patients with severe ADAMTS13 deficiency. Therefore, detailed medical history and complete work-up are recommended, including immunologic essays, such as (but not limited to) antinuclear, nucleosome, double-stranded and antiphospholipid Abs, C3 and C4 complement fragments, and screening for infections. Whenever possible, genetic testing is paramount to exclude cTTP [35,47,61]. Testing for immunoglobulin fractions can be useful in diagnosing CVID, though the association with iTTP is very rare, and only five other pediatric cases were reported [37].

All our patients exhibited the characteristic laboratory features of TMA, including severe thrombocytopenia, microangiopathic hemolytic anemia, elevated LDH, indirect hyperbilirubinemia, and schistocytosis. Reticulocytosis was present in all cases, supporting hemolysis-driven anemia and excluding mimicking conditions, such as vitamin B12 deficiency–related TMA. The clinical prediction scores proved valuable in all patients. The PLASMIC and French scores uniformly indicated a high probability of TTP, facilitating early treatment initiation prior to definitive ADAMTS13 confirmation. This was particularly important in Patients 2 and 4, in whom diagnostic uncertainty initially delayed referral. ADAMTS13 testing ultimately confirmed severe deficiency with detectable inhibitors in all cases. Serial assessment will be critical during follow-up, particularly in Patients 1 and 4, in whom the underlying triggers remain incompletely defined.

### 4.6. Therapeutic Agents

PEX remains the therapeutic backbone, with guideline documents emphasizing its role in removing pathogenic Abs and replenishing functional ADAMTS13 enzyme. PEX may be discontinued soon after a clinical response is achieved [56,62]. Usually, 1–1.5x plasma volume exchange is performed for the first three days, followed by 1x plasma volume exchange each day thereafter, while in patients with refractory TTP or evidence of progressive end organ damage, twice-daily PEX may be considered [31,39].

Adjunctive corticosteroids are routinely used to suppress autoAb production, and contemporary guidelines support initiating treatment as soon as possible when clinical suspicion is high, rather than awaiting confirmatory ADAMTS13 results [1,2]. Corticosteroids are usually associated with PEX as immunosuppressant drugs, and they may also reduce the number of procedures required and improve the tolerance of plasma therapy, despite the paucity of high-quality evidence-based studies [31,63]. Most standards of practice recommend oral prednisone 1 mg/kg/day or equivalent, with tapering over 3–4 weeks after clinical response is achieved; high-dose pulse steroids with 10 mg/kg/day for three days followed by 2.5 mg/kg/day thereafter may be more efficacious than 1 mg/kg/day dosing [39,52].

Caplacizumab, a vWF-targeting nanobody, has transformed early disease control by inhibiting platelet–vWF interaction, thereby reducing ongoing microvascular thrombosis. Adult randomized and real-world data consistently show faster platelet recovery and reduced exacerbation/relapse during treatment. Importantly for pediatric practice, multiple pediatric-focused reports, including case series and case reports, and pharmacologic dosing analysis describe its feasibility and apparent efficacy when integrated into standard iTTP regimens (PEX + immunosuppression), especially in high-risk presentations with severe thrombocytopenia and neurologic involvement [12,13,14,15,19,20]. Bleeding risk—predominantly mucocutaneous—remains a key safety consideration, and it is repeatedly emphasized that evaluating bleeding phenotypes in TTP is particularly relevant when caplacizumab is used in the setting of profound thrombocytopenia or invasive procedures [18,64,65]. These studies are primarily adult-focused; however, in pediatric practice, they provide a framework for discussing anticipated benefits (e.g., early disease control, fewer PEX procedures needed) and drawbacks (e.g., bleeding risk, cost). Pediatric dosing considerations and monitoring strategies are addressed in dedicated pediatric pharmacology work, supporting protocolized use under specialist supervision [14]. The doses used are of 10 mg daily for patients weighing > 40 kg and 5 mg for those weighing < 40 kg, for at least 30 days following the stop of PEX [34,47,52]. Health economic analyses further examine the budget and cost-effectiveness implications of adding caplacizumab to standard care from hospital and national perspectives, reinforcing that the drug’s clinical benefits must be interpreted. alongside resource use in systems where access may be constrained [64,66,67,68].

Rituximab is an approved immunomodulatory therapy in iTTP and is commonly employed to reduce autoAbs production, particularly in patients with high relapse risk, refractory disease, or as part of early combination strategies. When presenting pediatric versus adult data, it is appropriate to state that rituximab use is well established in adult settings; its use in children is largely by extrapolation and case-based evidence, with dosing and infection-risk considerations adapted to pediatric practice [30,31,32,69,70], largely through case series and narrative syntheses, with emphasis on relapse prevention and facilitating durable remission, while acknowledging the absence of large pediatric randomized trials [16,21,34,38]. The most frequently used dose is 375 mg/m^2^ weekly for four doses in total [39], with an expected 10-to-14-day delay to a documented effect [33].

Calcineurin inhibition (e.g., cyclosporine) and antimetabolite immunosuppression (e.g., mycophenolate mofetil) are often discussed in the iTTP literature as potential adjuncts in complex immune dysregulation, particularly when the therapeutic management is challenging; however, explicit pediatric evidence is limited. Therefore, any statements about these agents remain conservative: they are off-label agents considered case-by-case in complex immune contexts, typically after or combined with established therapies, with toxicity monitoring and infection prophylaxis tailored to age and comorbidity [30,31,32,46,71,72].

For multi-resistant or refractory iTTP, other off-label immunomodulatory strategies are increasingly described. Several agents are described in this setting. Bortezomib has been reported as a rescue therapy in rituximab-refractory iTTP, predominantly in adults, with multicenter experiences informing about the efficacy of its use when standard immunomodulation fails. Sustainable responses were registered in about 60% of multi-refractory iTTP patients with mild to moderate toxicities. Pediatric experience is limited but included in narrative syntheses and selected reports, underscoring the need for careful toxicity monitoring and specialist oversight [16,35,63,69]. Daratumumab (anti-CD38) is studied for efficacy and safety, representing another plasma cell-targeted salvage option. Although this evidence is primarily adult-based, it is directly relevant when discussing therapeutic escalation pathways and mechanisms of action in multiresistant iTTP [63,73]. Other anti-CD20 agents, such as obinutuzumab or ofatumumab, have been used in selected case [34]. These off-label immunosuppressive approaches are reported, but the dominant theme is individualized, salvage-oriented use, therefore requiring cautious interpretation [34,38,39].

Splenectomy can be considered in more severe patients with relapse/refractory iTTP who do not respond to other therapies [22,31,39].

Therapeutic PEX was promptly initiated in all patients after diagnosis. In Patient 1, more procedures were needed to achieve clinical response, but it is worth mentioning that she did not receive caplacizumab. Adjunctive corticosteroids were used to suppress ongoing autoAb production. In three out of four patients, low-dose methylprednisolone (1.5 mg/kg/day with slow tapering) was enough to obtain a response with minimal side effects.

The evolving pediatric experience with caplacizumab is particularly relevant to our cohort, as three patients presenting with high-risk features were treated with this new product. While long-term pediatric data remain limited, available evidence supports its use in severe presentations under expert supervision. There are still concerns regarding increased hospitalization costs when caplacizumab is used. Thus, obtaining the necessary approvals or the temporary unavailability of stock may pose challenges (as noted in Patients 1, 2 and 4). The improvement in disease management (fewer PEX procedures needed, quicker clinical response, reduced hospitalization period, and overall higher quality of life), as noticed in Patients 2, 3, and 4, compared to Patient 1, make caplacizumab an efficient new agent.

Rituximab represents a rational adjunct in patients with confirmed immune-mediated disease and high relapse risk, especially in the context of autoimmunity or immunodeficiency. As illustrated in Patients 1 and 3, Rituximab was employed in a combined strategy, due to lack of clinical response with the standard approach in autoimmunity settings. In Patients 2 and 4, Rituximab was added after each exacerbation of the disease.

Given the suspected/confirmed autoimmune trigger for iTTP in Patients 1 and 3, the addition of supplementary immunomodulation, namely mycophenolate mofetil, is supported by current evidence, especially after a challenging course of the disease; although, in an off-label setting, it was worth considering due to easy administration and few side effects.

### 4.7. Short- and Long-Term Outcomes

With modern therapy, short-term outcomes in iTTP have improved substantially [26,64,65], although severe cases with multiorgan failure continue to carry significant mortality risk [52]. Up to this point, proper treatment reduced mortality among patients from 90 to 10–20%; however, the recurrence rate is still high, reaching 36–50% of cases [30,55]. Pediatric series report high rates of hematologic remission when PEX is initiated promptly and combined with immunomodulation. However, outcomes are not limited to platelet recovery: acute neurologic events and complications related to vascular access or bleeding may shape early morbidity. In the caplacizumab era, pediatric reports suggest more rapid control of microvascular thrombosis and potentially fewer early exacerbations, although long-term pediatric data remain limited and heterogeneous [12,13,14,19,20,24].

Meticulous follow-up includes regular measurements of ADAMTS13 activity during the remission period. Some authors suggest measurements every trimester for a year, while others suggest longer-term follow-up. If a sudden drop in ADAMTS13 activity is experienced, rituximab treatment should be started to prevent a relapse [1,47].

Long-term outcomes in pediatric iTTP warrant dedicated attention because children have decades of life expectancy after an index episode. The adult literature describes persistent cognitive symptoms, fatigue, and reduced quality of life after TTP. On the other hand, pediatric-focused work emphasizes the importance of neurocognitive assessment, school functioning, and family-centered follow-up, alongside immunologic surveillance when autoimmunity is suspected, but not fully classifiable at onset. Relapse risk is also highlighted. Thus, the importance of planned follow-up and the need for surveillance of evolving autoimmune disease in secondary forms are highlighted [21,22,23,25,28,34,41,43,44,59,74]. The transition period from pediatric department to adult services is usually difficult and presents many challenges for many young adults with a history of iTTP. Therefore, transition programs are necessary [33].

All of the patients in this series require structured long-term follow-up, including platelets and hemoglobin levels, immunologic reassessment, and neurocognitive evaluation. Patients 1 and 4 warrant particular attention for potential evolution of underlying autoimmune disease, while Patient 2 requires coordinated immunology follow-up for CVID-related complications. Patient 3 illustrates the need for integrated rheumatologic and hematologic care in pediatric secondary iTTP associated with established SLE.

### 4.8. Complications

Complications span disease- and treatment-related domains. Disease-related complications include recurrent episodes (relapse/exacerbation), ischemic neurologic injury, renal dysfunction, and—more prominently in adults—cardiac ischemia. Treatment-related complications include line-associated infection or thrombosis, PEX-related hemodynamic instability, infections related to immunosuppression, hypogammaglobulinemia or serum sickness associated with rituximab, and bleeding, which is particularly relevant with caplacizumab. Cohorts presenting bleeding patterns in TTP provide an evidence base for risk communication and monitoring, supporting careful peri-procedural planning and individualized hemostatic strategies [2,18,25,28,52,65]. Secondary iTTP in autoimmune or infectious contexts introduces additional risks related to the underlying disease, imposing the need for multidisciplinary management [25,28]. Cytokine profiling studies and reviews emphasize that immune and inflammatory context may modulate susceptibility to complications and phenotypic variability, supporting careful individualized monitoring [30,31,32,49,51,66].

All our patients exhibited exacerbations of the disease, manifested by a decrease in platelet count (in Patients 1, 2 and 4) and/or a documented thrombotic event (in Patients 1 and 3), or a suspected transient ischemic cerebral attack (in Patient 4). The sudden drop in ADAMTS13 activity was a sign of impendent exacerbation in Patient 3, while central venous catheter acted as an additional risk factor for Patient 1. There was no relapse documented until the time of this paper. Notably, Patient 4 is currently under disease-directed therapy (low-dose corticosteroids).

Regarding treatment-related complications, to this date there have been no infections caused by central venous catheters or prolonged immunosuppressive therapy. Only in Patient 2 was minor bleeding was noted during caplacizumab treatment.

## 5. Conclusions

Pediatric secondary iTTP is a heterogeneous condition that frequently presents with diagnostic ambiguity and severe neurologic involvement. In a broader regional context, characterized by recurrent pediatric infectious challenges [75,76], maintaining a high index of suspicion for rare immune-mediated conditions such as pediatric iTTP remains essential.

The authors consider the biggest challenge regarding diagnostic procedures to be the turn-out time of ADAMTS13 testing (especially in medical facilities which do not have in-house tests); therefore, using the available scores can help with initiating treatment in a timely fashion. Moreover, these scores guide clinicians to consider iTTP (a rare pediatric pathology) as a possible diagnosis when faced with a patient with hemolytic anemia, severe thrombocytopenia, neurological symptoms and mild renal impairment. In order to decrease morbidity and mortality in this potentially life-threatening disease, it is paramount to have access to the most important therapeutic agents: PEX, corticosteroids, caplacizumab and rituximab. Therefore, early recognition, prompt initiation of TTP-directed therapy, and comprehensive immunologic evaluation are critical for favorable outcomes.

## Figures and Tables

**Table 1:** History, clinical data and laboratory results at diagnostic.

Category	Parameter	Patient 1	Patient 2	Patient 3	Patient 4
**Demographics**	Date of diagnosis	November 2021	September 2024	June 2025	December 2025
	Age (years)	14	14	17	11
	Sex	F	F	F	M
**Relevant** **history**	Major comorbidities/triggers *	None	Cellulitis of right tight after insect bite (2023)	Transient hemiparesis (2023)	None
**Clinical presentation**	Neurological symptoms	Vertigo, headache	Paresthesias, headache, agitation, slowed speech	Paresthesias, headache, severe agitation, facial palsy	Facial palsy, bradylalic
	Other symptoms	Fatigue, jaundice, vomiting	Jaundice	Palmar keratosis, high blood pressure, grade 3 obesity	Fatigue, abdominal pain, vomiting, gross hematuria, mild jaundice, overweight
**Hemolysis and cytopenias**	Hemoglobin (g/dL)	5.0	4.9	6.2	4.5
	Platelets (/mmc)	5000	27,000	8000	10,000
	MCV (/fl)	98	97.4	88.8	77.5
	Schistocytes	Present	Present	Present	Present
	Reticulocytes (/mmc)	450,000	350,000	370,000	200,000
	LDH (U/L)	2291	746	648	2074
	Haptoglobin (g/L)	0.08	0.08	0.1	0.06
	Total bilirubin (mg/dL)	3.4	2.3	2.8	2
	Indirect bilirubin (mg/dL)	2.4	1,4	1.9	1.4
**Coagulation/hemolysis workup**	INR	<1.5	<1.5	<1.5	<1.5
	Coombs test	Negative	Negative	Negative	Negative
**Renal and CNS involvement**	Creatinine (mg/dL)	0.96	0.85	1	0.74
	BUN (mg/dL)	45	28	67	73
	Brain imaging	Normal CT	Normal CT+MRI	Normal CT	Normal CT
**Clinical probability scores**	PLASMIC score	6	6	7	7
	French score	3	3	3	3
**Confirmatory testing**	ADAMTS13 activity	<1%	3.2%	<1%	<1%
	ADAMTS13 inhibitors (mixing studies)	Positive	Positive	Positive	Positive

* Infections, cancer, transplantation, medication, recent vaccination. F = female; M = male; MCV = mean corpuscular volume; INR = international normalized ratio; LDH = lactate dehydrogenase; CNS = central nervous system; BUN = blood urea nitrogen; CT = computer tomograph.

## Data Availability

Data is unavailable due to privacy. The raw data supporting the conclusions of this article will be made available by the authors on request.

## Abbreviations

The following abbreviations are used in this manuscript:

Ab(s)	antibody(ies)
AD	autosomal dominant
AR	autosomal recessive
CT	computer tomography
cTTP	congenital TTP
CVID	common variable immunodeficiency
DIC	disseminated intravascular coagulation
FRET	fluorescence resonance energy transfer
HUS	hemolytic uremic syndrome
HLA	human leukocyte alleles
IPF	immature platelet fraction
ITP	immune thrombocytopenic purpura
iTTP	immune-mediated TTP
INR	international normalized ratio
LDH	lactate dehydrogenase
MCV	mean corpuscular volume
MRI	magnetic resonance imaging
NGS	next-generation sequencing
PEX	plasma exchange
SLE	systemic lupus erythematosus
TMA	thrombotic microangiopathy
TTP	thrombotic thrombocytopenic purpura
vWF	von Willebrand factor
